# 全氟及多氟烷基物质非靶向识别中液相色谱-高分辨率质谱数据处理方法评价

**DOI:** 10.3724/SP.J.1123.2025.07011

**Published:** 2026-04-08

**Authors:** Boxuan ZHANG, Qinwen HE, Baocang HAN, Cundi MA, Zhujun LUO, Xiangzhou MENG

**Affiliations:** 1.同济大学环境科学与工程学院，上海 200092; 1. College of Environmental Science and Engineering，Tongji University，Shanghai 200092，China; 2.嘉兴同济环境研究院，浙江 嘉兴 314051; 2. Jiaxing Tongji Environmental Research Institute，Jiaxing 314051，China; 3.上海市公安局物证鉴定中心，上海 200083; 3. Institute of Forensic Science，Shanghai Municipal Public Security Bureau，Shanghai 200083，China

**Keywords:** 全氟及多氟烷基物质, 高分辨率质谱, 数据依赖性采集, 数据非依赖性采集, 峰提取, 解卷积, 非靶向识别, per- and polyfluoroalkyl substances （PFAS）, high-resolution mass spectrometry （HRMS）, data-dependent acquisition （DDA）, data-independent acquisition （DIA）, peak picking, deconvolution, nontarget identification

## Abstract

借助非靶向分析技术识别未知全氟及多氟烷基物质（per- and polyfluoroalkyl substances， PFAS）是环境领域的研究热点之一。液相色谱-高分辨率质谱是实施非靶向分析技术的主要手段。质谱数据的采集模式、峰提取算法及解卷积算法均会影响非靶向识别结果，但尚缺乏系统性评估。本研究基于超高效液相色谱-静电场轨道阱高分辨率质谱联用技术，通过基质加标样品，分别对比了两种软件（MS-DIAL和MZmine）的峰提取效果和两类算法（MS2Dec和IonDecon）的解卷积性能，评价了数据依赖性采集（DDA）与数据非依赖性采集（DIA）模式对PFAS非靶向识别结果的影响。结果表明，MS-DIAL的峰提取效果优于MZmine，检出全部34种目标物；针对DIA模式下的质谱数据，MS2Dec算法的解卷积效果优于IonDecon算法。DDA模式下识别结果的真阳性率随目标物浓度升高而提升，且阳性预测值保持在较高水平。相比之下，DIA模式下识别结果的真阳性率为100%，但其阳性预测值会随浓度升高而下降。最后，基于评价结果优化了DDA和DIA模式下的数据处理方法，并应用于3个实际电镀污泥样品检测，共鉴定出10类共计36种PFAS，其主要来源为电镀工艺中铬雾抑制剂的使用。本研究中的质谱数据处理方法可用于复杂环境基质中未知PFAS的识别。

## Abstract (en)

The widespread use， persistence， bioaccumulation， and toxicity of per- and polyfluoroalkyl substances （PFAS） have raised global concern. The number of PFAS types continues to grow， driven by changing industrial demands and regulatory environments. Non-target analysis using high-resolution mass spectrometry （HRMS） is an effective methodology for identifying novel and unknown PFAS in environmental matrices. The efficacy of non-target analysis is critically influenced by the data acquisition mode， peak picking algorithm， and deconvolution strategy. Using ultra-high performance liquid chromatography coupled with an Orbitrap mass spectrometer （UHPLC-Orbitrap MS）， this study aims to systematically evaluate data processing methods for non-targeted PFAS identification under data-dependent acquisition （DDA） and data-independent acquisition （DIA） modes. A clean sludge sample was spiked with 34 PFAS standards at three levels to assess method performance， alongside the analysis of three electroplating sludge samples. To compare the identification performance between DDA and DIA modes， a multi-step evaluation process was employed. Firstly， we assessed the peak picking capabilities of two widely used data processing software packages， MS-DIAL and MZmine. The key parameters for peak picking process are MS^1^ mass tolerance of 0.002 5 Da， MS^2^ mass tolerance of 0.01 Da， minimum peak height of 1 000， and retention time alignment tolerance of 0.1 min. Secondly， a comparison was made regarding DIA data deconvolution， specifically between MS2Dec algorithm and IonDecon algorithm. Finally， FluoroMatch was utilized to compare the true positive rate （TPR） and positive predictive value （PPV） of PFAS identification in both DDA and DIA datasets. In the spiked samples， the ［M-H］^-^ precursor ions for 33 PFAS standards and the ［M-CO_2_-H］^-^ ion for HFPO-DA were successfully detected and manually verified across all three levels. For peak picking， MS-DIAL demonstrated superior performance， achieving a 100% detection rate in all spiked samples， outperforming MZmine. When comparing deconvolution performance for DIA data， MS2Dec algorithm and the IonDecon algorithm showed similar efficacy， although MS2Dec algorithm exhibited slightly better results for low-concentration samples. In DDA mode， the true positive rate for PFAS identification increased from 80% to 100% with rising analyte concentration， accompanied by a minimal decrease in positive predictive value. Conversely， in DIA mode， the true positive rate remained at 100% across all concentrations， but positive predictive value decreased as concentration increased， primarily due to interferences from in-source fragmentation and adduct ions. The degree of in-source fragmentation of perfluorocarboxylic acids （PFCAs） decreases with increasing carbon chain length. However， the proportion of adduct ions remains nearly constant across different PFAS， leading to false positive identification of hydrogen-substituted PFAS. Based on the evaluation results， the data processing methods for DDA and DIA modes were optimized. These methods were then applied to three electroplating sludge samples， leading to the identification of 36 PFAS species belonging to 10 classes， including eight perfluorocarboxylic acids （PFCAs）， eight perfluorosulfonic acids （PFSAs）， one hydrogen-substituted perfluorosulfonic acid （H-PFSA）， five unsaturated perfluorosulfonic acids （UPFSAs）， one carbonyl perfluorosulfonic acid （KPFSA）， one chlorine-substituted perfluorosulfonic acid （Cl-PFSA）， one *n*∶2 fluorotelomer sulfonic acid （*n*∶2 FTSA）， five chlorinated polyfluoroethersulfonic acids （Cl-PFESAs）， two hydrogen-substituted polyfluoroethersulfonic acids （H-PFESAs）， and four polyfluoroethersulfonic acids （PFESAs）. Their presence was largely attributed to the use of chrome mist suppressants in the electroplating process. Combining DDA and DIA data for FluoroMatch input captured more information on unknown PFAS， possibly because the inclusion of multiple samples improves peak extraction. Based on the performance of PFAS identification in spiked and real samples， we developed a processing method that couples DDA and DIA data. This method can generate a composite list of identified PFAS while keeping data files independent， increasing the true positive rate and efficiency of identification. This study systematically evaluated nontargeted PFAS data processing methods， clarifying the optimal combination of tools for key steps （acquisition mode， peak picking， and deconvolution）， and validating its application potential in complex environmental matrices.

全氟及多氟烷基物质（per- and polyfluoroalkyl substances， PFAS）具有优异的热稳定性、化学惰性及疏水疏油特性，被广泛应用于消费品和工业领域^［1］^。然而，部分PFAS表现出环境持久性、生物累积性及潜在毒性，对生态系统和人类健康构成潜在风险^［2］^，已成为全球性关注议题。依据经济合作与发展组织（Organisation for Economic Co-operation and Development， OECD）的定义，分子结构中含有至少一个全氟化甲基（-CF_3_）或亚甲基（-CF_2_-）基团的化学物质均被归类为PFAS。目前，PubChem数据库中收录的此类化合物已逾七百万种^［3］^，且其数量持续增长。为发现环境中未知的PFAS，人们构建了非靶向识别技术，这弥补了靶向检测方法覆盖率有限的不足^［4］^。该技术主要依托高分辨率质谱（high-resolution mass spectrometry， HRMS）检测，并综合运用质量亏损过滤、同系物筛查、特征碎片离子匹配等多种数据处理与分析策略，以实现样品中PFAS的特征识别及结构解析^［5］^。

在高分辨率质谱驱动的PFAS非靶向识别中，数据采集模式的选择至关重要，直接决定了母离子（first stage of mass spectrometry， MS^1^）及二级质谱碎片（second stage of mass spectrometry， MS^2^）谱图信息的获取质量与范围。当前主流的采集策略包括数据依赖性采集（data-dependent acquisition， DDA）和数据非依赖性采集（data-independent acquisition， DIA）^［6］^。典型的DDA模式（如TopN）基于全扫描（full scan）数据，迭代选择丰度最高的前N个母离子进行碎裂。然而，该策略固有的丰度偏向性限制了低丰度母离子的碎裂及相应MS^2^谱图的获取。相较之下，DIA模式则对所有可检测的母离子进行碎裂，显著提升了对未知特征离子的MS^2^覆盖率。此外，DIA模式能够捕获DDA模式中通常缺失的关键信息，例如，低丰度碎片离子的同位素模式、二聚体/多聚体离子、各类加合物离子以及源内裂解产物的碎裂信息。在DIA模式下，由于母离子碎裂过程缺乏选择性，不同母离子产生的二级质谱碎片可能发生重叠。同时，共洗脱的PFAS分子及其他基质组分也可能产生相同质荷比的碎片离子，从而导致假阳性识别风险升高。

质谱峰提取与谱图解卷积也是非靶向识别流程中的关键步骤。该过程通过对高分辨率质谱数据的离子流信息进行提取、整合与关联，为后续结构识别提供可靠输入。Nason等^［7］^分别采用商用软件Compound Discoverer和开源平台FluoroMatch对土壤中未知PFAS进行识别，发现鉴定结果的主要差异源于峰提取阶段对PFAS特征离子的覆盖度，这一结论得到Jacob等^［8］^的证实。DIA模式数据的解卷积核心在于利用母离子（MS^1^）与其潜在二级质谱碎片（MS^2^）在色谱维度上的洗脱曲线（峰形）相似性，建立离子间的归属关系。例如，Koelmel等^［9］^应用基于离子流相关性的IonDecon算法对DIA数据进行解卷积，结果表明相较于DDA模式，DIA结合解卷积策略可将PFAS注释数量提升3倍以上。当前研究多倾向于使用集成化分析平台（如FluoroMatch^［10］^、FindPFΔS^［11］^、APP-ID^［12］^）提升PFAS非靶向识别效率。然而，尚未充分评估不同数据处理算法所致的特征离子信息的损失。优化峰提取与解卷积方法有助于最大限度捕获样品中存在的未知PFAS特征。

本研究基于超高效液相色谱-静电场轨道阱高分辨率质谱联用技术，旨在系统评价DDA和DIA两种模式下PFAS非靶向数据处理方法，比较不同方法对基质加标样品中PFAS特征峰的提取效率及解卷积效果。在此基础上，优化PFAS非靶向识别分析流程，并将其应用于实际样品（电镀污泥）中的PFAS非靶向筛查。此外，本研究还初步分析了PFAS化合物的源内裂解行为及加合离子形成规律。

## 1 实验部分

### 1.1 仪器、试剂与材料

Vanquish Flex超高效液相色谱仪-Orbitrap Exploris 240静电场轨道阱高分辨率质谱仪（美国Thermo Fisher公司）。

34种PFAS标准品和11种同位素内标均购自加拿大Wellington实验室，Pierce FlexMix校准溶液购自美国Thermo Fisher公司。甲醇（色谱纯）、氨水（色谱纯）和乙酸铵（纯度>99%）购自上海麦克林生化科技股份有限公司，Supelco ENVI-Carb固相萃取柱（250 mg，3 mL）购自德国Merck公司。

### 1.2 溶液的配制

PFAS标准使用液：准确移取250 μL质量浓度为2 μg/mL的34种PFAS标准品混合溶液至1.5 mL聚丙烯小瓶中，加入750 μL甲醇定容后混匀，即为质量浓度为500 μg/L的PFAS标准使用液，现用现配。

PFAS标准工作液：分别准确移取适量体积的500 μg/L的PFAS标准使用液至1.5 mL聚丙烯小瓶中，以甲醇为溶剂，配制质量浓度为50、100、200 μg/L的PFAS标准工作液。

同位素内标使用液：准确移取250 μL质量浓度为2 μg/mL的11种同位素内标混合溶液至1.5 mL聚丙烯小瓶中，加入750 μL甲醇定容后混匀，即为质量浓度为500 μg/L的同位素内标使用液，现用现配。

同位素内标工作液：准确移取400 μL质量浓度为500 μg/L的同位素内标使用液至1.5 mL聚丙烯小瓶中，加入600 μL甲醇定容后混匀，配制质量浓度为200 μg/L的同位素内标工作液。

### 1.3 样品制备与前处理

为评估质谱采集模式对PFAS非靶向识别效能的影响，并优化构建相应的数据处理方法，本研究采用基质加标样品进行方法开发与验证。加标样品基质取自温州某电镀园区污水处理厂污泥（经前期分析确证无PFAS污染）。基质经冷冻干燥（24 h）、研磨、过筛后，于-20 ℃保存备用。具体加标流程如下：分别称取3份0.5 g样品，依次加入5 μL质量浓度为50 μg/L、25 μL质量浓度为100 μg/L和125 μL质量浓度为200 μg/L的PFAS标准工作液，相应编号为SM1、SM2、SM3。为验证所构建数据处理流程的适用性，本研究将其应用于实际环境样品的PFAS非靶向分析。实际样品同样来源于温州某电镀园区污水处理厂，系处理不同类型电镀废水过程中产生的污泥（编号ES1、ES2、ES3）。样品经相同流程（冷冻干燥、研磨、过筛）处理后备用。

分别准确称取约0.5 g基质加标样品（SM1、SM2、SM3）及实际环境样品（ES1、ES2、ES3），置于离心管中，分别加入25 μL质量浓度为200 μg/L的同位素内标工作液，静置1 h。加入4 mL含0.2%（质量分数）氨水的甲醇溶液，经涡旋充分混匀后，于25 ℃下超声萃取15 min。随后以5 000 r/min离心15 min，收集上清液。重复此萃取步骤（加入4 mL氨水/甲醇溶液、涡旋、超声、离心、收集上清液）一次。继而加入4 mL含100 mmol/L乙酸铵的甲醇溶液，重复上述完整的提取流程（涡旋、超声、离心、收集上清液）。合并3次萃取所得上清液，在50 ℃、32 kPa条件下减压旋转浓缩至约1 mL。将浓缩液转移至预先经甲醇活化的Envi-Carb固相萃取（SPE）柱进行净化。以适量0.2%（质量分数）氨水甲醇淋洗SPE柱，收集淋洗液。合并的淋洗液在温和氮气流下吹扫浓缩至近干。残留物以甲醇溶解并定容至500 μL。所得溶液经0.22 μm尼龙滤膜过滤后，转移至进样瓶，于-20 ℃保存待测。

### 1.4 仪器分析

色谱条件 采用ACQUITY Premier BEH C18色谱柱（100 mm×2.1 mm， 1.7 μm）进行分离，柱温35 ℃。流动相组成如下：A相为含2 mmol/L乙酸铵的水溶液，B相为甲醇。洗脱梯度程序设定如下：0~1 min，5%B；1~3 min，5%B~70%B；3~10 min，70%B~95%B；10~12 min，95%B；12~12.1 min，95%B~5%B；12.1~15 min，5%B。流速恒定为0.3 mL/min，进样量为5.0 μL。

质谱条件 离子化采用电喷雾离子源，在负离子模式下运行。喷雾电压设置为-1.5 kV。离子传输管温度200 ℃，离子源雾化器温度350 ℃。鞘气（氮气）流速50 arb，辅助气（氮气）流速12.5 arb，吹扫气（氮气）流速0 arb。

### 1.5 数据采集与分析

本研究分别构建了基于DDA和DIA模式的数据处理方法，用于比较高分辨率质谱数据中PFAS的非靶向识别效果（图1）。

**图1:**
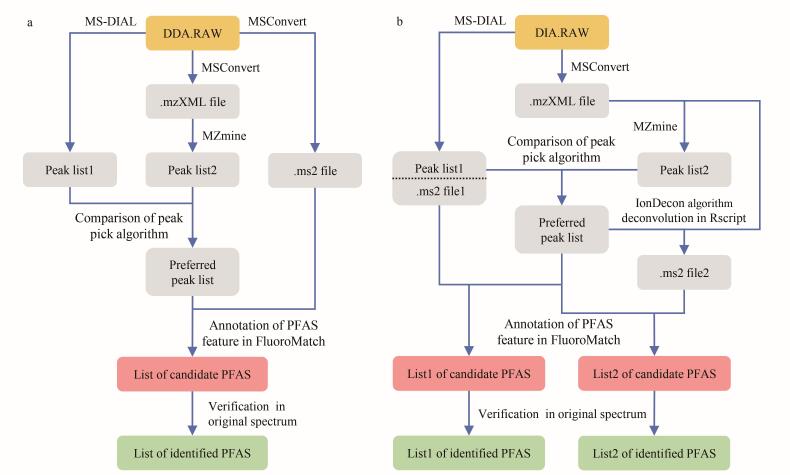
PFAS非靶向识别中（a）DDA模式与（b）DIA模式质谱数据处理方法

#### 1.5.1 DDA模式

采集模式 一级全扫描：分辨率120 000 FWHM，扫描范围*m/z* 80~1 000。射频透镜电压水平设为70%。自动增益控制模式为标准，最大注入时间设为自动。二级碎片离子扫描：基于一级扫描数据进行依赖性触发。分辨率15 000 FWHM，每周期选择丰度最高的前20个母离子（TopN=20）进行碎裂。隔离窗口宽度0.5 Da。采用阶梯式碰撞能量，分别为10、30和60 eV。扫描范围为自动设定。基质加标样品（SM1~SM3）及实际环境样品（ES1~ES3）在DDA模式下各进样分析一次。

峰提取与解卷积 原始数据（DDA.RAW）首先使用MS-DIAL（Version 5.5）直接进行峰提取，生成峰列表1（peak list1）。同时，使用MSConvert（Version 3.0）将原始数据转换为包含一级和二级质谱信息的mzXML文件（.mzXML file），随后导入MZmine（Version 2.26）进行峰提取，获得峰列表2（peak list2）。峰提取过程关键参数MS^1^质量容差为0.002 5 Da，二级质量容差为0.01 Da，最小峰高为1 000，保留时间对齐容差为0.1 min。对两份峰列表在34种目标PFAS特征离子的提取效能进行评估（基于检出数量），选择检出PFAS特征更多的一份峰列表（preferred peak list）用于后续分析。最后，利用MSConvert将原始数据转换为ms2格式文件（.ms2 file），并与优选出的峰列表一同导入FluoroMatch软件（Version 5.6），执行PFAS特征识别与结构鉴定。

特征识别与结构鉴定 基于样品的一级质谱精确质量数、二级质谱特征碎片离子、色谱保留时间、标准品匹配结果以及一级质谱同位素分布模式等信息，未知化合物的鉴定置信度被划分为1~5级^［13］^。针对PFAS，FluoroMatch开发者基于此置信度体系建立了PFAS分级规则^［14］^。等级A（可靠鉴定）需满足以下任一条件：（1）通过标准品确认结构（对应置信度1）；（2）依据精确质量数及PFAS特异性碎片离子推断分子结构（对应置信度2~3级）。等级B（推定鉴定）基于精确质量数及PFAS常见碎片离子进行结构推断，需进一步实验验证或人工审查确认（对应置信度3~5级）。本研究将等级A与等级B化合物纳入候选PFAS清单（list of candidate PFAS）。经复核原始谱图中的候选物同位素分布模式与碎片离子匹配性，并排除源内裂解产物及加合离子干扰后，最终将置信度为1~3级的PFAS判定为鉴定结果（list of identified PFAS）。

#### 1.5.2 DIA模式

采集模式 该模式包括两个扫描通道，通道1（一级全扫描）：分辨率120 000 FWHM，扫描范围*m/z* 80~1 000。其他参数设置同DDA模式的一级扫描。通道2（二级碎片离子扫描）：非依赖性触发，进行全范围碎裂，分辨率15 000 FWHM。采用宽隔离窗口，宽度100 Da。扫描范围为自动设定。隔离窗口间设置5 Da的质量重叠以确保覆盖连续性。其余二级参数设置同DDA模式。基质加标样品（SM1~SM3）及实际环境样品（ES1~ES3）在DIA模式下各进样分析一次。

峰提取与解卷积 利用与DDA采集模式相同的方法进行峰提取，获得优选特征峰列表（preferred peak list）。随后，分别实施两种解卷积路径：（1）使用MS-DIAL的MS2Dec算法直接对原始数据（DIA.RAW）进行解卷积处理^［15］^，生成ms2格式文件1（.ms2 file1），关键参数Sigma窗口值为0.7；（2）使用MSConvert将原始数据转换为mzXML格式文件（.mzXML file），再将该文件与优选峰列表（preferred peak list）共同输入含有IonDecon算法的R语言脚本进行解卷积处理^［9］^，生成ms2格式文件2（.ms2 file2），关键参数峰相关系数为0.7。

特征识别与结构鉴定 将上述优选峰列表分别与解卷积结果1和解卷积结果2输入FluoroMatch软件，进行PFAS的特征识别与结构鉴定，获得候选PFAS清单（list1 of candidate PFAS和list2 of candidate PFAS）。经复核原始谱图中的候选物同位素分布模式与碎片离子匹配性，并排除源内裂解产物及加合离子干扰后，分别获得基于两种解卷积算法（MS2Dec与IonDecon）识别的PFAS列表（list1 of identified PFAS和list2 of identified PFAS），用于后续方法学比较。

#### 1.5.3 评价方法

为了评价不同采集模式下PFAS非靶向识别结果，本文使用了真阳性率（true positive rate， TPR）和阳性预测值（positive predictive value， PPV）两个指标^［8］^。真阳性率衡量了非靶向识别技术识别样品中存在的PFAS特征的能力，反映了该技术的灵敏度。阳性预测值衡量了非靶向识别过程避免对样品中不存在的PFAS特征进行错误注释的能力，反映了该技术的准确性。

TPR=TP/（TP+FN）（1）

PPV=TP/(TP+FP)（2）

在非靶向识别中，真阳性（TP）是指：一个实际存在于样品中的化合物（或特征）被正确识别并报告的结果；假阴性（FN）是指：一个实际存在于样品中的化合物（或特征）被错误报告为不存在；假阳性（FP）是指：一个实际不存在样品中的化合物（或特征）被错误地报告为存在。

### 1.6 质量控制与质量保证

样品采集、运输、前处理及仪器分析全程禁止使用含聚四氟乙烯（PTFE）材质的器材。实验过程中设置了现场空白、实验程序空白和仪器溶剂空白进行污染监控。所有样品均制备3份平行样。为最大程度降低管路背景污染与吸附效应，仪器流路系统采用聚醚醚酮（PEEK）及不锈钢材质。分析前使用标准校准液对质量检测器进行校准，确保质量偏差控制在<10^-6^（<1 ppm）。系统适用性测试要求进样序列前、中、后时段注入的50 ng/mL PFAS标准工作液中，目标分析物峰面积的相对标准偏差（RSD）<10%。基质加标样品中同位素标记内标的峰面积RSD应<20%。针对非靶向数据处理，仅选取实际样品中信号强度（峰面积）超过空白样品对应信号5倍的特征峰进行后续分析。

## 2 结果与讨论

### 2.1 非靶向识别效果

#### 2.1.1 峰提取

使用Xcalibur软件对原始数据进行手动识别，确定了34种PFAS及10种同位素内标在高分辨率质谱中的分析结果（表1）。在3个基质加标样品（SM1、SM2、SM3）中均检出33种PFAS的［M-H］^-^母离子峰以及HFPO-DA的［M-CO_2_-H］^-^离子峰；而HFPO-DA的［M-H］^-^离子峰仅在SM2和SM3样品中出现。这是因为HFPO-DA属于全氟醚羧酸（perfluoroalkyl ether carboxylic acid，PFECA），在质谱分析中极易发生源内裂解，导致其［M-H］^-^母离子在离子源内分解成碎片^［16］^。本研究的质谱分析温度（离子传输管200 ℃和雾化器350 ℃）在PFAS的分子离子响应和源内裂解控制两方面取得了平衡^［17］^，但高于专门分析PFECA类物质的温度设置（离子传输管150 ℃和雾化器200 ℃）^［18］^。这种较高的温度会进一步削弱PFECA类物质［M-H］^-^母离子的响应强度，导致其在低浓度样品中离子峰的缺失。

**表1:** 基质加标样品中PFAS信息、保留时间及质谱参数

Compound	Abbr.	Formula	Ion type	Theoretical exact mass （*m/z*）	Measured accurate mass （*m/z*）	Retention time/min	Mass error/10^-6^（ppm）
Perfluorobutanoic acid	PFBA	C_4_HF_7_O_2_	［M-H］^-^	212.97920	212.97920	4.88	0.00
Perfluoropentanoic acid	PFPeA	C_5_HF_9_O_2_	［M-H］^-^	262.97601	262.97614	5.33	0.49
Perfluorohexanoic acid	PFHxA	C_6_HF_11_O_2_	［M-H］^-^	312.97281	312.97294	5.74	0.42
Perfluoroheptanoic acid	PFHpA	C_7_HF_13_O_2_	［M-H］^-^	362.96962	362.96916	6.19	1.27
Perfluorooctanoic acid	PFOA	C_8_HF_15_O_2_	［M-H］^-^	412.96643	412.96620	6.73	0.55
Perfluorononanoic acid	PFNA	C_9_HF_17_O_2_	［M-H］^-^	462.96323	462.96325	7.29	0.04
Perfluorodecanoic acid	PFDA	C_10_HF_19_O_2_	［M-H］^-^	512.96004	512.96020	7.84	0.32
Perfluoroundecanoic acid	PFUndA	C_11_HF_21_O_2_	［M-H］^-^	562.95684	562.95696	8.38	0.21
Perfluorododecanoic acid	PFDoA	C_12_HF_23_O_2_	［M-H］^-^	612.95365	612.95374	8.87	0.15
Perfluorotridecanoic acid	PFTrDA	C_13_HF_25_O_2_	［M-H］^-^	662.95046	662.95079	9.32	0.50
Perfluorotetradecanoic acid	PFTeDA	C_14_HF_27_O_2_	［M-H］^-^	712.94726	712.94746	9.71	0.28
Perfluoro-2-methyl-3-oxahexanoic acid	HFPO-DA	C_6_HF_11_O_3_	［M-H］^-^	328.96773	328.96777	5.85	0.12
4，8-Dioxa-3*H*-perfluorononanoic acid	NaDONA	C_7_H_2_F_12_O_4_	［M-H］^-^	376.96887	376.96847	6.22	1.06
2*H*，2*H*-Perfluorooctanoic acid	6∶2 FTCA	C_8_H_3_F_13_O_2_	［M-H］^-^	376.98527	376.98483	6.38	1.17
2*H*，2*H*-Perfluorodecanoic acid	8∶2 FTCA	C_10_H_3_F_17_O_2_	［M-H］^-^	476.97888	476.97876	7.52	0.25
2*H*，2*H*-Perfluorododecanoic acid	10∶2 FTCA	C_12_H_3_F_21_O_2_	［M-H］^-^	576.97249	576.97272	8.63	0.40
Perfluorobutanesulfonic acid	PFBS	C_4_HF_9_O_3_S	［M-H］^-^	298.94299	298.94285	5.38	0.47
Perfluoropentanesulfonic acid	PFPeS	C_5_HF_11_O_3_S	［M-H］^-^	348.93980	348.93918	5.76	1.77
Perfluorohexanesulfonic acid	PFHxS	C_6_HF_13_O_3_S	［M-H］^-^	398.93660	398.93606	6.21	1.36
Perfluoroheptanesulfonic acid	PFHpS	C_7_HF_15_O_3_S	［M-H］^-^	448.93341	448.93278	6.73	1.41
Perfluorooctanesulfonic acid	PFOS	C_8_HF_17_O_3_S	［M-H］^-^	498.93022	498.92957	7.27	1.30
Perfluorononanesulfonic acid	PFNS	C_9_HF_19_O_3_S	［M-H］^-^	548.92702	548.92683	7.82	0.35
Perfluorodecanesulfonic acid	PFDS	C_10_HF_21_O_3_S	［M-H］^-^	598.92383	598.92415	8.35	0.53
Perfluoro-4-ethylcyclohexane	PFECHS	C_8_HF_15_O_3_S	［M-H］^-^	460.93341	460.93286	6.64	1.19
1*H*，1*H*，2*H*，2*H*-Perfluorohexane sulfonic acid	4∶2 FTSA	C_6_H_5_F_9_O_3_S	［M-H］^-^	326.97429	326.97424	5.70	0.15
1*H*，1*H*，2*H*，2*H*-Perfluorooctane sulfonic acid	6∶2 FTSA	C_8_H_5_F_13_O_3_S	［M-H］^-^	426.96790	426.96759	6.73	0.73
1*H*，1*H*，2*H*，2*H*-Perfluorodecane sulfonic acid	8∶2 FTSA	C_10_H_5_F_17_O_3_S	［M-H］^-^	526.96152	526.96155	7.88	0.06
9-Chlorohexadecafluoro-3-oxanonane-1-sulfonic acid	6∶2 Cl-PFESA	C_8_HF_16_ClO_4_S	［M-H］^-^	530.89558	530.89514	7.47	0.83
11-Chloroeicosafluoro-3-oxaundecane-1-sulfonic acid	8∶2 Cl-PFESA	C_10_HF_20_ClO_4_S	［M-H］^-^	630.88919	630.88940	8.49	0.33
Perfluorobutane sulfonamide	FBSA	C_4_H_2_F_9_SNO_2_	［M-H］^-^	297.95898	297.95868	6.03	1.01
Perfluorohexane sulfonamide	FHxSA	C_6_H_2_F_13_SNO_2_	［M-H］^-^	397.95259	397.95197	7.23	1.56
Perfluorooctane sulfonamide	FOSA	C_8_H_2_F_17_SNO_2_	［M-H］^-^	497.94620	497.94577	8.51	0.86
*N*-Methyl perfluorooctanesulfonamidoacetic acid	*N*-MeFOSAA	C_11_H_6_F_17_NO_4_S	［M-H］^-^	569.96733	569.96753	8.13	0.35
*N*-Ethyl perfluorooctanesulfonamidoacetic acid	*N*-EtFOSAA	C_12_H_8_F_17_NO_4_S	［M-H］^-^	583.98298	583.98309	8.38	0.19
Perfluoro-*n*-（^13^C_4_）butanoic acid	^13^C_4_-PFBA	^13^C_4_HF_7_O_2_	［M-H］^-^	216.99262	216.99263	4.88	0.05
Perfluoro-*n*-（1，2-^13^C_2_）pentanoic acid	^13^C_2_-PFHxA	^13^C_2_^12^C_4_HF_11_O_2_	［M-H］^-^	314.97952	314.97961	5.74	0.29
Perfluoro-*n*-（1，2，3，4-^13^C_4_）octanoic acid	^13^C_4_-PFOA	^13^C_4_^12^C_4_HF_15_O_2_	［M-H］^-^	416.97984	416.97955	6.73	0.70
Perfluoro-*n*-（1，2，3，4，5-^13^C_5_）nonanoic acid	^13^C_5_-PFNA	^13^C_5_^12^C_4_HF_17_O_2_	［M-H］^-^	467.98001	467.97977	7.29	0.51
Perfluoro-*n*-（1，2-^13^C_2_）decanoic acid	^13^C_2_-PFDA	^13^C_2_^12^C_8_HF_19_O_2_	［M-H］^-^	514.96675	514.96667	7.84	0.16
Perfluoro-*n*-（1，2-^13^C_2_）undecanoic acid	^13^C_2_-PFUnDA	^13^C_2_^12^C_9_HF_21_O_2_	［M-H］^-^	564.96355	564.96362	8.38	0.12
Perfluoro-*n*-（1，2-^13^C_2_）dodecanoic acid	^13^C_2_-PFDoA	^13^C_2_^12^C_10_HF_23_O_2_	［M-H］^-^	614.96036	614.96008	8.87	0.46
Perfluoro-1-hexane（^18^O_2_）sulfonic acid	^18^O_2_-PFHxS	C_6_HF_13_^18^O_2_^16^OS	［M-H］^-^	402.94510	402.94455	6.21	1.36
Perfluoro-1-（1，2，3，4-^13^C_4_）octanesulfonic acid	^13^C_4_-PFOS	^13^C_4_^12^C_4_HF_17_O_3_S	［M-H］^-^	502.94364	502.94348	7.27	0.32
1*H*，1*H*，2*H*，2*H*-Perfluoro（1，2-^13^C_2_）octane sulfonic acid	^13^C_2_-6∶2 FTSA	^13^C_2_^12^C_6_H_5_F_13_O_3_S	［M-H］^-^	428.97461	428.97440	6.73	0.49
1*H*，1*H*，2*H*，2*H*-Perfluoro（1，2-^13^C_2_）decane sulfonic acid	^13^C_2_-8∶2 FTSA	^13^C_2_^12^C_8_H_5_F_17_O_3_S	［M-H］^-^	528.96823	528.96828	7.88	0.09

本研究比较了MS-DIAL和MZmine对PFAS标准物质离子峰的检测率，以及DDA和DIA采集模式下二级质谱图的生成率（图2）。在3个基质加标样品中，MS-DIAL均成功识别出全部34种PFAS的特征峰，其峰提取能力优于MZmine。该结果与文献［^19^］结论一致：MS-DIAL的峰提取结果与人工核查吻合度最高。MZmine作为FluoroMatch工作流的默认配置^［10］^，实际分析中研究者常直接采用软件或脚本的默认参数提取特征峰，但缺乏对算法特征覆盖率的系统验证。

**图2:**
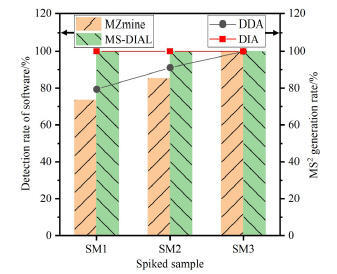
DDA模式与DIA模式下目标PFAS的峰检出率及其MS^2^生成率

采用高覆盖率算法可显著降低化合物漏检风险，从而提升非靶向识别的整体识别效能。DDA模式下采集的二级质谱信息通常少于DIA模式，特别是在低浓度样品中（SM1）。随着目标PFAS浓度升高，DDA模式下的二级质谱生成率从80%提升至100%，而DIA模式下的二级质谱生成率均为100%，Li等^［20］^也发现了类似的变化趋势，表明化合物浓度是影响DDA采集效果的关键因素。

#### 2.1.2 解卷积算法

图3对比了DIA数据（分别由IonDecon与MS2Dec算法解卷积）与DDA数据的PFAS非靶向识别的真阳性率（TPR）与阳性预测值（PPV）。两种解卷积算法的TPR与PPV表现相近，但在SM1样品中MS-DIAL解卷积效果更优。DIA模式在3个基质加标样品中的TPR均为100%，但其阳性预测值随样品中PFAS浓度升高呈现下降趋势。主要原因是高浓度样品在离子源内产生更为复杂的源内裂解碎片和加合离子，解卷积算法可能将其误判为独立的化合物，导致假阳性识别增加。

**图3:**
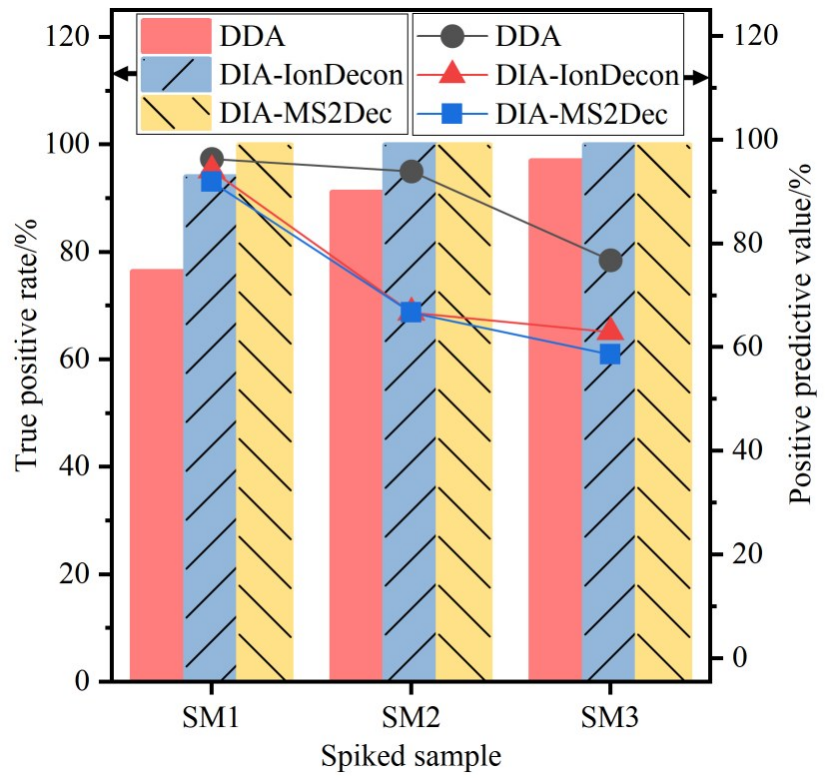
DDA模式与DIA模式下目标PFAS的真阳性率和阳性预测值

尽管DIA模式在单位保留时间窗口内产生较高通量的共洗脱碎片，致使其解卷积生成的二级质谱图质量普遍低于DDA模式^［6］^，但对基于碎片匹配的识别方法影响有限（TPR未显著降低）。而对于依赖谱图相似性比对的筛选方法^［21，22］^，则需对二级质谱进行严格过滤^［20］^。相比之下，DDA模式虽具有较高阳性预测值，但TPR均低于两种解卷积算法下的DIA数据。化合物浓度是影响DDA模式识别效率的关键因素，即使在高浓度条件下，目标化合物的碎片生成率仍可能受非目标特征干扰和质谱采集随机性抑制，导致谱图获取不全。

#### 2.1.3 源内裂解与加合离子

源内裂解和加合离子现象诱导单一母体PFAS产生多重碎片峰，形成“伪分子离子簇”，显著增加自动特征提取与后续数据分析的复杂性。部分能量被消耗于碎片离子生成，真实分子离子［M-H］^-^的丰度随之降低，极端情况下甚至完全消失，导致母体化合物被误判为假阴性（如HFPO-DA的漏检）。图4展示了3类羧酸PFAS母离子（［M-H］^-^）与常见源内裂解离子（［M-CO_2_-H］^-^和［M-CF_2_O-H］^-^）及加合离子（［M+CH_3_COONa-H］^-^）的占比分布^［17，23］^。值得注意的是，同一化合物在不同浓度中维持高度一致的离子组分比例。随着碳链长度增加，［M-CO_2_-H］^-^离子占比持续下降，这归因于CF_2_-CO_2_^-^键解离能随链长升高^［17］^，造成源内裂解程度与链长呈负相关。

**图4:**
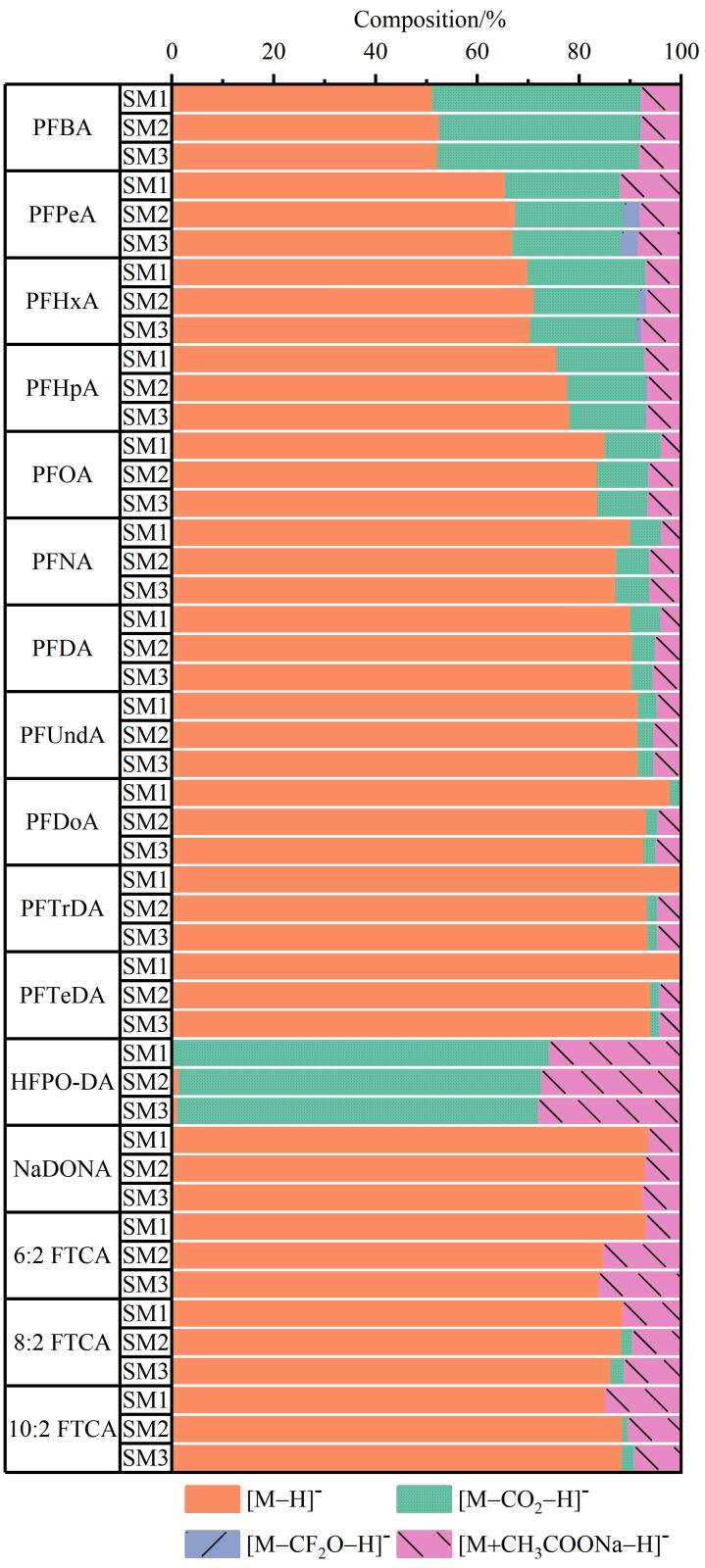
羧酸类PFAS母离子、源内裂解离子和加合离子的峰面积占比

相较于文献［^24^］条件，本研究采用更低的离子源电压与温度，有效抑制了源内裂解作用，故［M-CF_2_O-H］^-^仅在较高浓度样品中微量检出。而加合离子［M+CH_3_COONa-H］^-^在所有样品中保持恒定占比（约10%），证明了流动相与基质效应对分子离子检测的显著影响。需要特别注意，［CH_3_COONa］（*m/z* 82.003 07）与氢取代PFAS碎片［C_2_HF_3_］^ -^（*m/z* 82.003 03）的质量数接近，易导致［M+CH_3_COONa-H］^-^型离子被错误识别为氢取代PFAS离子峰^［23，25］^。通过系统检查不同PFAS特征的保留时间，可有效降低此类氢取代PFAS的假阳性识别风险^［26］^。

### 2.2 实际样品PFAS非靶向识别

#### 2.2.1 DDA模式与DIA模式差异

基于2.1节的评估结果，使用MS-DIAL软件对实际样品的DDA和DIA模式采集的数据进行峰提取和解卷积，并利用FluoroMatch软件对电镀污泥样品进行PFAS非靶向识别。图5展示了DDA与DIA模式下污泥样品中PFAS的识别结果。受限于特定样品的基质特性，两种模式下识别出的PFAS数量未见显著差异。在整体识别比例上，DIA模式表现出更高的特征离子覆盖率，凸显了其在复杂体系中化合物特征识别方面的技术优势^［6］^。FluoroMatch软件采用基于碎片离子匹配的算法进行结构解析。DIA模式对所有母离子进行全碎裂，导致解卷积过程中可用于匹配的二级谱图信息显著增加，从而大幅提高了单个化合物获得有效碎片匹配的概率。与单独处理DDA模式或DIA模式下采集的数据相比，将两种采集模式的数据整合并导入FluoroMatch软件，可识别出更多潜在的PFAS特征。这主要源于低丰度特征离子易受响应强度波动影响，导致峰形不规则。多批次样品间的峰对齐处理可有效校正此类峰形畸变，提升峰积分精度。另外，通过纳入同一样品的多次进样或同批次检测多个样品数据进行联合识别可显著提升识别结果的可靠性^［27］^。

**图5:**
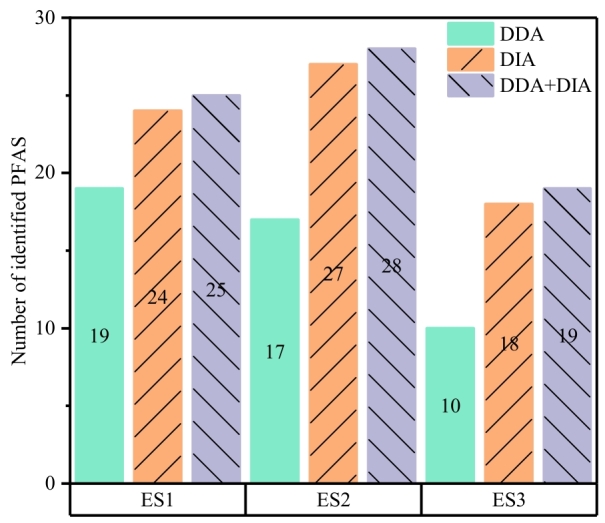
实际样品（电镀污泥）中PFAS非靶向识别数量

基于加标样品和实际样品中PFAS识别的效果，我们建立了耦合DDA和DIA数据的处理方法，如图6所示。将DDA和DIA数据同时导入MS-DIAL进行峰提取得到峰列表。使用MSConvert将DDA数据转化为ms2文件1，使用MS-DIAL对DIA数据进行解卷积得到ms2文件2。将上述得到的峰列表和所有ms2文件一起导入FluoroMatch进行PFAS识别，该方法可在保持文件数据独立的情况下获得复合的识别PFAS清单，提高识别真阳性率和效率。

**图6:**
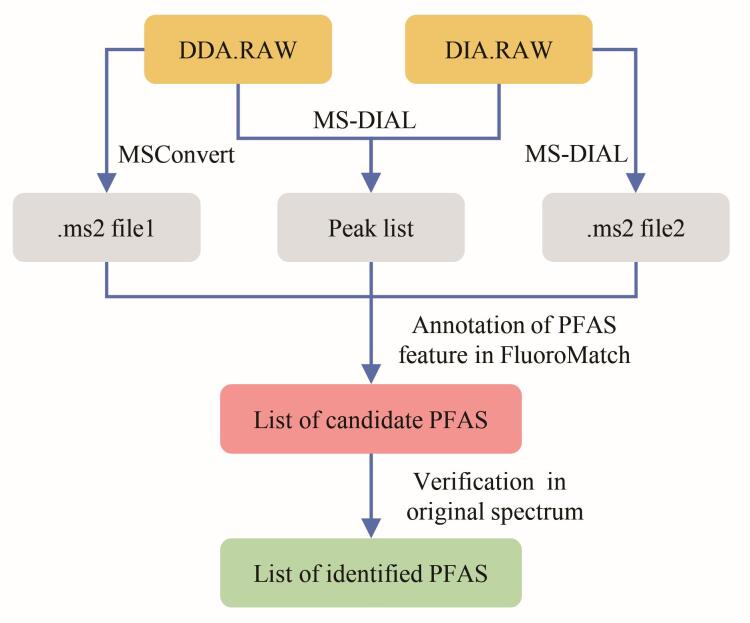
PFAS非靶向识别中质谱数据处理优化方法

#### 2.2.2 非靶向PFAS检出情况

从3个样品中共识别出10类36种PFAS，包括8种全氟羧酸（PFCA）、8种全氟磺酸（PFSA）、1种氢取代全氟磺酸（H-PFSA）、5种不饱和全氟磺酸（UPFSA）、1种羰基全氟磺酸（KPFSA）、1种氯取代全氟磺酸（Cl-PFSA）、1种*n*∶2氟调聚磺酸（*n*∶2 FTSA）、5种氯代多氟醚磺酸（Cl-PFESA）、2种氢取代多氟醚磺酸（H-PFESA）和4种多氟醚磺酸（PFESA）（见表2）。其中，H-PFSA、UPFSA、KPFSA和Cl-PFSA作为消防泡沫中PFSA的副产物或杂质被检出^［28］^。在电镀行业，这些物质更可能来源于以全氟辛烷磺酸（PFOS）为主要成分的铬雾抑制剂^［29］^。H-PFESA通常被视为Cl-PFESA的合成副产物或环境转化产物^［30，31］^，而PFESA类物质则可能作为Cl-PFESA的替代品用于铬雾抑制剂配方中^［29］^。本次非靶向识别出17种目标PFAS，其中大部分已在电镀生产环节广泛报道^［32，33］^，其余非目标PFAS的检出结果具有重要的环境意义，亟需予以充分重视，以便更准确地评估PFAS的环境赋存状况及其潜在生态与健康风险。

**表2:** 实际样品（电镀污泥）中非靶向识别的PFAS信息、保留时间及质谱参数

Compound class	Abbr.	Formula	Theoretical exact mass （*m/z*）	Measured accurate mass （*m/z*）	Retention time/min	Mass error/10^-6^ （ppm）
Perfluorocarboxylic acid	PFCA	C_3_HF_5_O_2_	162.98240	162.98228	2.88	0.74
		C_4_HF_7_O_2_	212.97920	212.97929	4.89	0.42
		C_5_HF_9_O_2_	262.97601	262.97597	5.36	0.15
		C_6_HF_11_O_2_	312.97281	312.97296	5.74	0.48
		C_7_HF_13_O_2_	362.96962	362.96927	6.18	0.96
		C_8_HF_15_O_2_	412.96643	412.96603	6.73	0.96
		C_10_HF_19_O_2_	512.96004	512.96021	7.77	0.33
		C_11_HF_21_O_2_	562.95684	562.95728	8.26	0.78
Perfluorosulfonic acid	PFSA	C_4_HF_9_O_3_S	298.94299	298.94324	5.42	0.84
		C_5_HF_11_O_3_S	348.93980	348.93918	5.75	1.77
		C_6_HF_13_O_3_S	398.93660	398.93606	6.18	1.36
		C_7_HF_15_O_3_S	448.93341	448.93278	6.73	1.41
		C_8_HF_17_O_3_S	498.93022	498.92957	7.22	1.30
		C_9_HF_19_O_3_S	548.92702	548.92683	7.78	0.35
		C_10_HF_21_O_3_S	598.92383	598.92415	8.26	0.53
		C_11_HF_23_O_3_S	648.92064	648.92060	8.76	0.06
Hydrogen-substituted perfluorosulfonic acid	H-PFSA	C_8_H_2_F_16_SO_3_	480.93964	480.93940	7.03	0.50
Unsaturated perfluorosulfonic acid	UPFSA	C_7_HF_13_SO_3_	410.93660	410.93661	6.21	0.02
		C_8_HF_15_SO_3_	460.93341	460.93301	6.63	0.87
		C_9_HF_17_SO_3_	510.93022	510.93011	7.11	0.22
		C_10_HF_19_SO_3_	560.92702	560.92720	7.59	0.32
		C_11_HF_21_SO_3_	610.92383	610.92384	7.96	0.02
Carbonyl perfluorosulfonic acid	KPFSA	C_8_HF_15_SO_4_	476.92833	476.92812	6.80	0.44
Chlorine-substituted perfluorosulfonic acid	Cl-PFSA	C_8_HF_16_ClSO_3_	514.90067	514.90100	7.17	0.64
*n*∶2 fluorotelomer sulfonic acid	*n*∶2 FTSA	C_8_H_5_F_13_SO_3_	426.96790	426.96716	6.62	1.73
Chlorinated polyfluoroethersulfonic acid	Cl-PFESA	C_7_HF_14_ClSO_4_	480.89878	480.89886	7.01	0.17
		C_8_HF_16_ClSO_4_	530.89558	530.89514	7.47	0.83
		C_10_HF_20_ClSO_4_	630.88919	630.88940	8.49	0.33
		C_11_HF_22_ClSO_4_	680.88600	680.88519	8.84	1.19
		C_12_HF_24_ClSO_4_	730.88281	730.88342	9.33	0.83
Hydrogen-substituted polyfluoroethersulfonic acid	H-PFESA	C_8_H_2_F_16_SO_4_	496.93455	496.93442	6.41	0.26
		C_10_H_2_F_20_SO_4_	596.92817	596.92828	7.33	0.18
Polyfluoroethersulfonic acid	PFESA	C_7_HF_15_SO_4_	464.92833	464.92822	6.85	0.24
		C_8_HF_17_SO_4_	514.92513	514.92487	7.31	0.50
		C_9_HF_19_SO_4_	564.92194	564.92200	7.76	0.11
		C_11_HF_23_SO_4_	664.91555	664.91571	8.55	0.24

## 3 结论

本研究基于液相色谱-高分辨率质谱的DDA和DIA采集模式数据，系统比较了不同峰提取方法及解卷积算法的性能，确立了最优分析流程。该方法在DIA模式下实现了加标样品中所有目标PFAS的全面识别。进一步研究发现，融合DDA与DIA数据进行非靶向分析，可有效提升未知PFAS的发现能力。应用优化方法对实际电镀污泥样品进行分析，共识别出10类36种PFAS，包括28种磺酸类PFAS和8种羧酸类PFAS。分析表明，其主要来源于电镀工艺中铬雾抑制剂的使用。本研究为环境介质PFAS的识别提供了可靠分析策略，有助于支撑我国新污染物治理工作。
